# Genetic Dissimilarity between Mates, but Not Male Heterozygosity, Influences Divorce in Schistosomes

**DOI:** 10.1371/journal.pone.0003328

**Published:** 2008-10-08

**Authors:** Sophie Beltran, Frank Cézilly, Jérôme Boissier

**Affiliations:** 1 Laboratoire de Biologie et d'Ecologie Tropicale et Méditerranéenne, UMR 5244 CNRS-EPHE-UPVD, Université de Perpignan, Perpignan, France; 2 Equipe Ecologie Evolutive, UMR CNRS 5561 Biogéosciences, Université de Bourgogne, Dijon, France; The University of New South Wales, Australia

## Abstract

**Background:**

Correlational studies strongly suggest that both genetic similarity and heterozygosity can influence female mate choice. However, the influence of each variable has usually been tested independently, although similarity and heterozygosity might be correlated. We experimentally determined the relative influence of genetic similarity and heterozygosity in divorce and re-mating in the monogamous endoparasite *Schistosoma mansoni*.

**Methodology/Principal Findings:**

We performed sequential infections of vertebrate hosts with controlled larval populations of parasites, where sex and individual genetic diversity and similarity were predetermined before infection. Divorce rate increased significantly when females were given the opportunity to increase genetic dissimilarity through re-mating with a new partner, independently of the intensity of male-male competition. We found however no evidence for females attempting to maximize the level of heterozygosity of their reproductive partner through divorce.

**Conclusions/Significance:**

Female preference for genetically dissimilar males should result in more heterozygous offspring. Because genetic heterozygosity might partly determine the ability of parasites to counter host resistance, adaptive divorce could be an important factor in the evolutionary arms race between schistosomes and their hosts.

## Introduction

Both heterozygosity and genetic similarity have been predicted to influence female mate preferences. According to the good-genes-as-heterozygosity hypothesis [1], [2], females should choose mates with a high level of overall heterozygosity. Mating with such males would be beneficial for females because highly heterozygous males should produce offspring which are more heterozygous than the offspring of males chosen at random. This hypothesis predicts that given a choice between two males, females should prefer the more heterozygous one. Alternatively, the disassortative mating hypothesis [3] states that females should prefer to mate with genetically dissimilar males in order to produce offspring with high levels of heterozygosity. This hypothesis differs from the previous one in emphasizing the importance of male and female genotypes in determining offspring fitness. The disassortative mating hypothesis predicts that given a choice between two males, females should choose the one which is the most genetically dissimilar to themselves. Although these hypotheses have attracted a large attention, most studies to date are correlational, tend to focus on one effect in isolation, and are biased towards vertebrate species. Recent evidence for an inter-correlation between these two forms of genetic quality [4] suggests however that both kinds of effect should be simultaneously studied in analyses of mate choice [5]–[7].

Direct evidence of adaptive female mate choice based on genetic quality can be difficult to obtain. First, evidence for an effect of genetic quality on mate choice critically depends on the availability of a sufficient number of genetic markers [8], as a large number of loci is likely to give more accurate estimates of genome-wide individual heterozygosity and genetic dissimilarity between mates [9]. Second, evidence for adaptive mate choice critically relies on the possibility of complete sampling of all mating alternatives, what is often difficult to obtain in the field. Studies of re-mating provide an interesting alternative, however [10]. Divorce can be considered as an extension of mate choice after initial pairing [11], and comparisons between the qualities of old and new mates may then provide a reliable cue as to which factor influences mate choice. Although a large literature exists on divorce in monogamous bird species [12], [13], [14], little is known about the causes and consequences of divorce in other monogamous species, especially among invertebrates.

We investigated the influence of genetic dissimilarity and heterozygosity on re-mating in the endoparasite *Schistosoma mansoni*. *S. mansoni* is unique in the sense that it uses both sexual reproduction in the vertebrate definitive host and asexual multiplication in the mollusc intermediate host. The sexual reproduction ensures the production of new genotypes, whereas the asexual multiplication enables genotype proliferation (i.e. clonal expansion). We were therefore able to perform experimental infections of vertebrate hosts with controlled clonal populations, with sex and individual genetic diversity being predetermined before infection.

**Figure 1:**
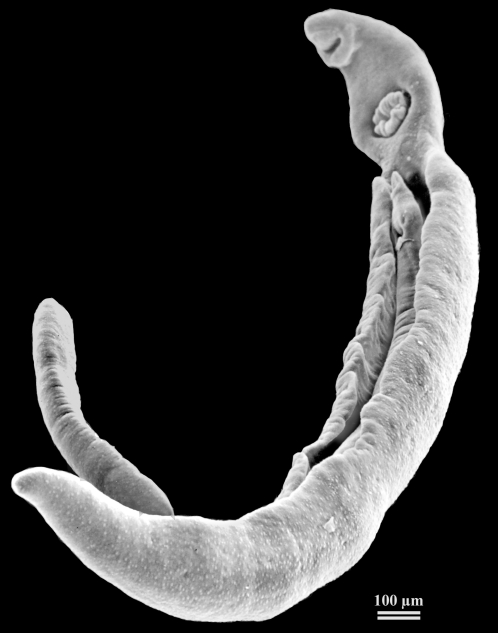
A schistosome pair, with the thin female located in the male gynaecophorical canal.

Schistosomes are an ideal biological model to test the influence of genetic quality on re-mating. Indeed, the mating system of *Schistosoma mansoni* is best described as sequential monogamy [15], [16]. Pairs form in the vertebrate definitive host, with the muscular male keeping its thin female in a groove on its ventral side called the gynaecophorical canal (Fig. 1). Divorce has been previously observed in *S. mansoni* [15], although its functional significance remains elusive.

## Results

### Experiment 1

Previous studies have shown that divorce regularly occurs in *S. mansoni*, but did not provide any information about the proximate factors promoting divorce. One important factor might be the male-biased sex ratio that is typically observed in schistosome infrapopulations [17]. A male-biased sex ratio may promote male-male competition and, hence, “forced” divorce, i.e. displacement of a paired male by an unpaired rival [11], [12], [18], but also favour female choice of a better mating option [11], [12], [19]. Likewise, divorce might also be facilitated by a female-biased sex-ratio (although this is unlikely to occur under natural conditions) through female-female competition and/or males abandoning their current mate for a better mating option. We therefore conducted a first series of experiments to assess whether divorce rate was equally likely to occur whether the sex-ratio was male- or female-biased.

**Figure 2:**
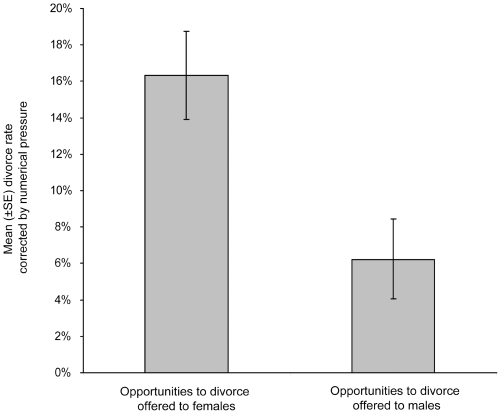
Differences in mean divorce rate corrected by numerical ‘pressure’ (±SE), according to which sex is given opportunities to divorce. The numerical ‘pressure’ is measured as the number of individuals introduced during the second infection divided by the number of individual of the same sex introduced on the first one.

To that end, we performed multiple infections of definitive hosts with a first infection using parasites of both sexes followed by either male or female re-infection. Divorce rate per host ranged between 33% and 50% when mice were re-infected with males (experiment 1a), whereas it varied between 0% and 11% when mice were re-infected with females (experiment 1b) (permutation test for two independant samples, *m* = *n* = 4, *P* = 0.0143). After correcting for the numerical ‘pressure’ (i.e. the male-male or female-female competition measured as the number of individuals introduced during the second infection divided by the number of individual of the same sex introduced on the first one), divorce rate per host ranged between 10% and 20% when mice were re-infected with males (experiment 1a) whereas it varied between 0% and 9% when mice were re-infected with females (experiment 1b). However, the difference between the two treatments remained significant after correcting for the numerical ‘pressure’ (Fig 2; permutation test for two independent samples, *m* = *n* = 4, *P* = 0.0143).

### Experiment 2

A second series of multiple infections, following the same procedure as the used in experiment 1 and using a single different clonal population for each sex, was performed to assess the relative influence of both male heterozygosity and genetic dissimilarity between mates on divorce rate. Given the results obtained in the first series of experiments, we used a male-biased sex ratio (experiment 1a), thus providing females with opportunities to re-mate with newcomers male of a different genotype than that of their first mate. For each combination of one female and one male clonal population, two or three different male genotypes were independently offered as alternative mating options. Newcomer males could be either less or more heterozygous and less or more genetically dissimilar to females than their first mates.

**Figure 3:**
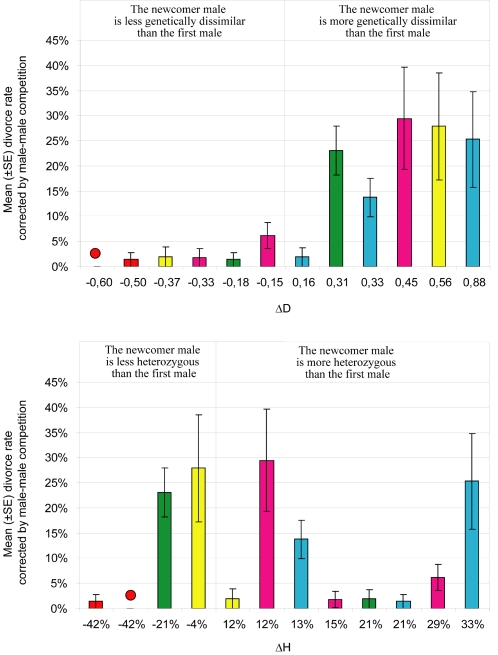
Influence of genetic dissimilarity between mates and male heterozygosity on divorce rate corrected by male-male competition in schistosomes. Genetic dissimilarity was estimated using the r coefficient of Wang [20] and heterozygosity was calculated for each male clone as the number of heterozygous loci divided by the total number of loci. We defined ΔH as the difference in overall heterozygosity between the newcomer male and the first male, and ΔD as the difference in genetic dissimilarity to the female between the first male and the newcomer male (Table 1). When positive, ΔH and ΔD indicate increased heterozygosity in females' progeny. Each colour represents a different female clone. The intensity of male-male competition is measured as the number of individuals introduced during the second infection divided by the number of individual of the same sex introduced on the first one. A total of 528 pairs was analysed.

Overall, five different female clones and nine different male clones were used in the experiments (Table 1). ΔD varied between −0.60 to 0.88, while ΔH varied between −42% to +33%. Divorce rate, corrected by the intensity of male-male competition, ranged from 0 to 29% (Fig. 3), and differed significantly between tests (logistic regression, *χ^2^* = 98.11, d.f. = 11, *P*<0.0001). Results from a logistic regression indicated that genetic dissimilarity had the larger influence on divorce rate (*χ^2^* = 24.79, d.f. = 1, p<0.0001, whereas the intensity of male-male competition (*χ^2^* = 5.44, d.f. = 1, p = 0.020) and female clone (*χ^2^* = 10.16, d.f. = 4, p = 0.038) had only a marginal effect. By contrast, male heterozygosity had no significant influence on divorce rate (*χ^2^* = 0.64, d.f. = 1, p = 0.424). Overall, females were more likely to switch mates when the newcomer male was more dissimilar to themselves than their first mate. When ΔD was positive, the mean divorce frequency corrected by the intensity of male-male competition (20.2%±4.3%) was about ten times higher than for negative values of ΔD (2.11%±0.9%).

**Table 1:** Experimental protocol used in tests of divorce.

	First male heterozygosity	Newcomer male heterozygosity	ΔH	Genetic similarity between the female and the first male	Genetic similarity between the female and the newcomer male	ΔD	Number of replicates
Female 1	0.58	0.70	0.12	0.394	0.763	−0.369	4
	0.58	0.55	−0.03	0.394	−0.169	0.563	4
Female 2	0.42	0.63	0.21	0.235	0.075	0.160	4
	0.42	0.55	0.13	0.235	−0.094	0.329	4
	0.42	0.75	0.33	0.235	−0.645	0.880	4
Female 3	0.55	0.67	0.12	−0.094	−0.543	0.449	3*
	0.55	0.83	0.28	−0.094	0.056	−0.150	4
	0.55	0.70	0.15	−0.094	0.235	−0.329	3*
Female 4	0.63	0.83	0.20	−0.135	0.049	−0.184	4
	0.63	0.42	−0.21	−0.135	−0.443	0.308–	4
Female 5	0.83	0.42	−0.42	−0.288	0.212	−0.500	4
	0.83	0.42	−0.42	−0.288	0.309	−0.597	3*

Male heterozygosity was calculated for each clone as the number of heterozygous loci divided by the total number of loci. Genetic similarity was estimated using the r coefficient of Wang [20]. ΔH represents the difference between the heterozygosity of the newcomer male and the heterozygosity of the first male. ΔD represents the difference between the genetic similarity between the first male and the female and the genetic similarity between the newcomer male and the female.

* indicates that one mouse died before the end of the experiment.

## Discussion

Male and female schistosomes were assumed to form stable monogamous pairs until it was shown that divorce could occur among pairs of *S. mansoni* in vivo [15]. However, until now, the adaptive function of divorce in schistosomes remained elusive. Only a few studies have used molecular markers to infer mating patterns in parasite species [21]. Although it has been suggested that *S. mansoni* paired randomly according to their genetic relatedness [22], the empirical evidence was based on paired individuals recovered from only three rats, and failed to take into account the genotype of unpaired individuals present inside the definitive host [23]. In the present study, divorce rate was significantly higher when the sex-ratio was experimentally male-biased rather than female-biased. In addition, genetic dissimilarity, but not male heterozygosity, had a positive influence on divorce rate.

Basically, divorce in schistosomes could come about in two different ways [12]: i) one partner may abandon the other or ii) one partner may be chased by an usurper. The role of each sex in either case might depend on the costs associated with divorce. Results from the first experiment suggest both that males rarely initiate divorce, even when alternative mating options are available, and that the role of female-female competition is negligible. This might be explained by the fact that the female can only grow and mature once she has paired with a male [17]. One direct consequence of that is that pairing is particularly costly for males in terms of energy and nutrients transferred to the female during her growth. Such a large investment would irremediably be lost by males who initiate divorce, thus limiting their interest in doing so. A second consequence is that immature females of tiny size are probably unable to dislodge larger, paired females. We are thus left with the possibility that male-male competition is the main cause of divorce, or with the possibility that female directly initiate divorce when they perceive a better mating option [11], [12], [19]. Abandoning a male might be costly to a female too as she may regress to an immature stage. However, regression to an immature state takes from 3 to 6 days [24], a period of time that might be sufficient to pair with another male, especially under a male-biased sex-ratio.

Contrary to previous observations [15], the intensity of male-male competition (i.e. the number of males from the second infection over the number of males from the first infection in each mouse in our second series of experiments) had only a marginal influence on divorce rate in the present study. This seems contrary to what is expected if mate change correspond to “forced divorce”[18], [25], [26], i.e. paired female being taken over by single males. By contrast, genetic dissimilarity had a strong influence on divorce rate, suggesting that monogamous female schistosomes enhance outbreeding by divorcing for males that are less related to themselves than their first mate. A comparable phenomenon has been previously observed in the monogamous long-tailed tit, *Aegithalos caudatus*, where optimal outbreeding provided a better explanation than the “forced divorce” hypothesis to patterns of mate-switching [26]. It is however possible that both the “forced divorce” hypothesis and the “better option” hypothesis contribute to explain our results. Indeed the behaviour of females may influence the success of take-over attempts. Depending on the perceived quality of the usurper, female may choose to help its current mate to resist a take-over attempt or not, a form of passive female choice [27], [28]. The relevance of passive female choice in schistosomes remains to be assessed though direct observations *in vitro*.

While female preference for genetic dissimilarity is likely to result in increased offspring heterozygosity, male mating success does not appear to be dependent on individual heterozygosity at microsatellite loci since differences in heterozygosity between males had no influence on the rate of divorce. We are confident that the absence of effect of heterozygosity was not due to an insuficient number of microsatellite loci [8]. First, we used 12 microsatellite loci with a satisfactory average level of polymorphism, whereas most studies investigating mate choice based on microsatellite heterozygosity have used between five to nine loci [7], [8]. Second, an insufficient number of genetic markers would have equally affected our ability to estimate genetic dissimilarity between mates. The highly significant and strong effect of genetic dissimilarity between mates on the divorce rate suggests however that we used a large enough number of microsatellites.

So far, the influence of female choice on re-mating has been essentially studied in monogamous bird species [11], [13]. Our results provide for the first time evidence of adaptive divorce in an invertebrate monogamous species. Female preference for genetically dissimilar males may contribute to explain both the heterozygous excess found in both male and female schistosome natural populations [29], [30], and the fact that mean relatedness measured between male and female pairs in vertebrate hosts is always negative [29]. It may also have important consequences in terms of genetic population structure and transmission strategy of the parasite. Schistosomes cause a serious human disease called schistosomiasis. Schistosomiasis ranks second only to malaria in terms of parasite-induced human morbidity and mortality, with over 200 million people infected and 600 million at risk worldwide [31]. Because genetic heterozygosity might partly determine the ability of parasites to counter host resistance, adaptive divorce could be an important factor in the evolution of drug resistant strains of *Schistosoma* [32].

## Materials and Methods

### Parasite Life cycle

*Schistosoma mansoni* is a human and rodent parasite. Parasite sexual reproduction occurs between male and female adult worms in the vertebrate definitive host. Sex determination is syngamic, i.e. one egg produces either one male or one female larva (called miracidium). The aquatic larva actively infects a mollusc intermediate host from *Biomphalaria* genus, transforms into intramolluscan larval stages (called sporocysts) and produces, by clonal multiplication, many unisexual larvae (called cercariae) that will actively infect the vertebrate definitive host and transform into adult worms. In this study, we used a *Schistosoma mansoni* strain isolated in December 2004 from naturally infected molluscs from Guadeloupe (French West Indies), a Guadeloupean strain of *Biomphalaria glabrata* as intermediate hosts and the Swiss OF1 mouse strain as final hosts. Methods for mollusc, mouse infections and parasite recovery have been previously described [30].

### Heterozygosity and genetic dissimilarity estimations

Heterozygosity was calculated for each clone as the number of heterozygous loci divided by the total number of loci, and genetic dissimilarity was estimated using the r coefficient of Wang [20], which considers the probability for two individuals to share an allele, given the estimated allele frequencies in the population. In the present study, the population considered consisted of all clonal populations obtained following mollusc infection. To compute the r coefficient of Wang we check for the absence of both departure from Hardy-Weinberg distribution and linkage disequilibria using Genepop version 3.4 [33]. The r coefficient was then calculated using the program SPAGeDi 1.0 [34]. We defined ΔH as the difference in overall heterozygosity between the newcomer male (or female) and the first mate and ΔD as the difference in genetic similarity to the female (or to the male) between the first mate and the newcomer male (or the newcomer female). When ΔH or ΔD negative, the newcomer sexual partner is less heterozygous or less genetically dissimilar than the first one. At the opposite, when ΔH or ΔD positive, the newcomer sexual partner is more heterozygous or more genetically dissimilar than the first male.

### Experiment 1: Initiating sex on divorce

#### Production, sexing and genotyping parasite clones

Four hundred and fifty six molluscs were individually exposed to one miracidia. Five weeks later, from these 456 exposed molluscs, 39 were infected and emitted cercariae. Two cercariae of each infected mollusc were sexed and genotyped using W1 marker, a female specific sequence only present on the W chromosome [35] and 10 microsatellite markers, R95529, Smd57, SmD28, L46951 [36], SmC1, SmDO11[37], SmBr8, SmBr13, SmBr14, SMBr16 [38]. From the 39 infected molluscs, 18 emitted female clonal cercarial populations, and 21 emitted male clonal cercarial populations.

#### Experimental protocol

A first group of four mice (experiment 1a) were individually infected, using 20 female cercariae from a single clone and 30 male cercariae from a single clone. The excess of males ensured that all females would have been mated. At five weeks post infection, mice were infected again with 45 cercariae from another clonal population of males. This provided females with opportunities to re-mate with newcomer males of a different genotype than that of their first mate. The different clonal populations used in this first experiment were chosen to avoid any influence of genetic dissimilarity and/or heterozygosity on either male or female divorce rate. The genetic dissimilarity between the female and its first mate, and between the female and its second mate was 0.2345 and 0.0655 respectively (ΔD = 0.1690). The number of heterozygous (6/10) loci did not differ between the first and second male (ΔH = 0). A second group of four mice (experiment 1b) were individually infected using 30 female cercariae from a single clone and 20 male cercariae from a single clone. The excess of females ensured that all males would have been mated. At five weeks post infection, mice were infected again with 45 cercariae from another clonal population of females. This provided males with opportunities to re-mate with newcomer females of a different genotype than that of their first mate. The genetic dissimilarity between the male and its first mate, and between the male and its second mate was 0.3585 and 0.1343 respectively (ΔD = 0.2242). The number of heterozygous loci (7/10) did not differ between the first and the second female (ΔH = 0). The paired and unpaired male of the first group and female of the second group were isolated and counted. The DNA of all these worms was extracted and the microsatellite profile was determined.

### Experiment 2: Relative influence of male heterozygosity and genetic dissimilarity between mates on female choice to re-mate

#### Production, sexing and genotyping parasite clones

Four hundred and thirty six molluscs were individually exposed to one miracidium. Five weeks later, from these 436 exposed molluscs, 37 were infected because they emitted cercariae. Two cercariae of each infected mollusc were sexed and genotyped using W1 marker [35] and 12 microsatellite markers, R95529, SmD57, SCMSMOXII, SmD89, SMIMP25[36], SmC1, SmDO11 [37], SmBR8, SmBR13, SmBR14, SmBR16 [38], SMS7 [39]. From the 37 infected molluscs, 17 emitted female clonal cercarial populations, and 20 emitted male clonal cercarial populations.

#### Experimental protocol

Tests of divorce were performed in five separate series of tests. Each test consisted in individually infecting four mice using 45 male cercariae from a single clone and 30 female cercariae from a single clone. The excess of males ensured that all females would be mated after four weeks. Indeed, one control group of three mice infected using 45 male cercariae from a single clone and 30 female cercariae from a single clone showed that all females were mated after four weeks. At five weeks post infection, each mouse was independently infected again with 67 cercariae of a new male clonal population genetically distinct from that of the first male. Each female clone was tested with two or three newcomer male clones, with various genetic dissimilarities with the female and various levels of heterozygosity (i.e. different ΔH and ΔD) (Table 1). Seven weeks after the second infection, all mice were sacrificed and worms were recovered [40]. From all the re-infected mice, the paired and unpaired adult worms were isolated and counted. The DNA of all male worms was extracted and their microsatellite profile was determined.

#### Parameters measured and statistical analyses

Divorce rate was measured as the number of re-mated pairs on the total number of pairs. The intensity of competition (equivalent to the availability of alternative mating options to females), was measured as the number of individuals introduced during the second infection divided by the number of individual of the same sex introduced on the first one. As re-mating was defined as a binomial variable, the influence of male-male competition, male heterozygosity, and genetic dissimilarity on divorce rate, was analysed using a logistic regression model, controlling for female genotype.

#### Ethical note

Our laboratory has received the permit N° A 66040 for experiments on animals from both French Ministère de l'Agriculture et de la Pêche and French Ministère de l'Education Nationale de la Recherche et de la Technologie. Housing, breeding and animal care of the mice followed the ethical requirements of our country. The experimenter possesses the official certificate for animal experimentation delivered by both ministries (Décret n° 87–848 du 19 octobre 1987; number of the authorization 007083).
